# Migraine management: Non-pharmacological points for patients and health care professionals

**DOI:** 10.1515/med-2022-0598

**Published:** 2022-11-23

**Authors:** Faraidoon Haghdoost, Mansoureh Togha

**Affiliations:** Headache Department, Iranian Center of Neurological Research, Neuroscience Institute, Tehran, Iran; The George Institute for Global Health, University of New South Wales, Sydney, Australia; Headache Department, Neurology Ward, Sina Hospital, School of Medicine, Tehran University of Medical Sciences, Tehran, Iran

**Keywords:** migraine, non-pharmacological, prevention, trigger factors

## Abstract

Migraine is a highly prevalent disorder with an enormous burden on societies. Different types of medications are used for controlling both acute attacks and prevention. This article reviews some non-pharmacological recommendations aiming to manage migraine disorder better and prevent headache attacks. Different triggers of migraine headache attacks, including environmental factors, sleep pattern changes, diet, physical activity, stress and anxiety, some medications, and hormonal changes, are discussed. It is advised that they be identified and managed. Patients should learn the skills to cope with the trigger factors that are difficult to avoid. In addition, weight control, management of migraine comorbidities, lifestyle modification, behavioural treatment and biofeedback, patient education, using headache diaries, and improving patients’ knowledge about the disease are recommended to be parts of migraine management. In addition, using neuromodulation techniques, dietary supplements such as riboflavin, coenzyme Q10 and magnesium, and acupuncture can be helpful. Non-pharmacological approaches should be considered in migraine management. Furthermore, the combination of pharmacological and non-pharmacological approaches is more effective than using each separately.

## 1 Introduction

Migraine is a common headache disorder and one of the most disabling disorders globally. It is responsible for more than 50% of all years lost due to neurological disorder-related disability and is the first cause of disability in under the 50 s. Migraine significantly impacts social, physical, and occupational function [1,2,3,4]. Its global prevalence in males, and females, is 9.8%, and 18.9%, respectively (overall prevalence of 14.4%). The incidence of migraine peaks at 35–45 years, and although the prevalence is similar in girls and boys before puberty, the female to male ratio is estimated to be three to one in reproductive ages [4,5,6,7,8]. Although migraine is associated with a considerable burden, it can be well controlled using pharmacological and non-pharmacological approaches.

Due to the impact of migraine on society and patients, appropriate management and treatment should be considered. Mild attacks are treated by analgesics and antiemetics, while ergotamine, dihydroergotamine, and triptans are added for moderate to severe attacks [9,10]. For several reasons such as poor response to acute treatment, adverse effects of abortive medication, the risk of overuse of acute medications, contraindications to some abortive drugs in some migraine patients, disabling attacks, patient preference, and high frequency of attacks, prophylactic treatment might be necessary using various drugs, including beta-blockers, calcium channel blockers, antidepressants, antiepileptics, and anti-calcitonin gene-related peptide (CGRP) drugs [11,12]. On the other hand, some drugs are associated with adverse effects, and patients are concerned about the side effects. Furthermore, medication overuse headache (MOH), a condition of daily headache because of acute drug overuse, high economic burdens of medications, limited effects of abortive and prophylactic drugs, decreased tendency to take the medication in some patients, pregnancy and lactation, and contraindications of some preventative medications might be other reasons for seeking preventive and non-pharmacological strategies [13,14,15,16]. The focus of migraine management should be shifted from medication alone to adding alternative and non-pharmacological approaches to overcome the mentioned concerns and achieve better results. The benefits of non-pharmacological migraine management are listed in Table 1.

**Table 1:** Benefits of non-pharmacological migraine management

– Avoiding or reducing the adverse events of medications
– Reducing the economic burden of medications
– Reducing the chance of MOH
– Helping individuals with migraine who do not want to take medications
– Helping individuals with migraine who do not get expected results from medications
– Treating migraine during pregnancy and lactation to avoid drug therapy
– Treating migraine when there are contraindications to some medications
– Increasing the efficacy of abortive and preventive treatments

Different non-pharmacological approaches are suggested for migraine management. Behavioural therapy and biofeedback, education, relaxation, mindfulness, and weight reduction are among the mentioned techniques [17,18,19,20]. Other recommendations are considering lifestyle modifications such as having proper sleep and exercise, regular meals, and stress management [21]. As many physiological and psychological triggers can provoke migraine attacks, considering them in the successful long-term management of migraine is crucial [22]. In addition, some non-pharmacological interventions, including neuromodulation techniques, dietary supplements, such as riboflavin, coenzyme Q10 and magnesium, and acupuncture, are used for the management of migraine [23]. Additionally, it is worth mentioning that a combination of pharmacological and non-pharmacological approaches is more effective than using each separately [13,14,24].

According to the mentioned benefits of non-pharmacological approaches for migraine management, this article reviews non-pharmacological recommendations and strategies aiming at better management of migraine disorder and prevention of headache attacks.

## 2 Migraine diagnosis

Migraine is diagnosed based on the patient’s history using proposed criteria by the International Classification of Headache Disorders 3rd Edition (ICHD-III) [25]. For adults, diagnosis is based on the following criteria: at least five untreated headache attacks lasting 4–72 h with at least two of the following characteristics: unilateral location, pulsatile quality, moderate to severe intensity, and aggravation by routine physical activities. Each attack must be associated with nausea (and/or vomiting), and/or photophobia, and phonophobia. Approximately 20% of individuals with migraine experience migraine with aura. Aura is a sensory, visual, or other central nervous system symptom followed by headache attacks. Aura can sometimes happen after the beginning of a headache attack or continue during the headache phase. In some cases, the aura may occur independent of the headache [13,25].

Migraine includes different phases (premonitory, aura, headache attack, and postdrome), and patients may experience all or some of them in their headache attacks. The premonitory phase, which is defined as the presence of a non-specific symptom, such as changes in mood or appetite or a higher sensory sensitivity, is more common than aura and can be considered a warning as it occurs hours or a day or two before headache attacks. While the headache subsides gradually (resolution phase), patients may experience fatigue, food intolerance, irritability, and mood changes, defined as the postdrome phase [26].

## 3 Management of/coping with migraine trigger factors

Migraine attacks can be initiated by several extrinsic and intrinsic factors. Identifying and avoiding or limiting exposure to these factors is part of non-pharmacological prevention of migraine. Exposure to these factors may predict the probability of a migraine headache, which provides people with migraine with a valuable opportunity to take action when they are susceptible to an attack. In addition, those who have migraine will develop a sense of control over the disorder by being informed about potential triggers. Furthermore, patients must be educated that headaches may start hours after exposure to a trigger factor, depending on the trigger type. For example, the mean time to the onset of an attack is about 22 h after chocolate consumption and around 3 h after drinking red wine [27,28,29].

Common migraine trigger factors are summarised in Table 2. It is important to note that trigger factors are different from risk factors, and the terms should not be used for the same purpose. Female gender, positive family history of migraine, and low socioeconomic status are among the risk factors for developing migraine; however, trigger factors induce migraine attacks in patients with migraine. Several reasons are mentioned for the mechanisms behind trigger factors, such as a decreased threshold of migraine (e.g. by oestrogen withdrawal in menstruation), induction of cortical spreading depression (e.g. due to mild head trauma) and activation of trigeminal nociceptors in the meninges [13,26,27,28,29,30]. The effect of trigger factors is different from one patient to another, and differences in vulnerability to the factors are not well recognised between migraine with aura and migraine without aura [29]. According to the additive view of migraine triggers, a combination of trigger factors may initiate a headache attack, and patients should be aware of the attack provocation when a combination occurs. For instance, working without eating during menstruation is an important combination. Although some triggers, such as menstruation, are thought to be singly sufficient to provoke a migraine attack, combinations are associated with a higher risk of headache attacks and should be considered [31]. Triggers can act individually or in combination to impact the outcome. This can be different from person to person according to the concept of neural representations (neurotags) and pain perception [32,33].

**Table 2:** Common migraine trigger factors

Migraine trigger factors
Stress/relaxation following stress/anxiety
Physical activity
Fatigue/prolonged overexertion/sexual activity
Sleep
Lack/too much/too little/irregular pattern/changes
Hormonal factors
Menstruation/menopausal fluctuations
Environment
High altitude
Heat
Weather changes
Loud sound
Flashing lights/bright light/sunlight
Sharp blow to head
Changes in barometric pressure
Odours
Strong odours/perfumes/fumes/smoke
Medication
Nitrates/theophylline/reserpine/nifedipine/indomethacin/cimetidine/oral contraceptives/vasodilators
Dietary
Fasting/hunger/skipped meal
Dehydration
Ice-cream/ice-water
Aged cheese/nuts/onions
Chocolate
Wine/beer/alcohol
Foods containing monosodium glutamate/tyramine/nitrate
Caffeine overuse
Infections
Upper respiratory infections
Cervical spine problems
Prolonged fixed position of neck
Discopathy
Cervical muscle spasm
Temporomandibular joint disorders
Parafunctional
Malocclusion
inflammation

All migraine patients cannot avoid the whole list of trigger factors. Prioritising the trigger factors among the general migraine population and identifying the triggers in every patient individually regarding their impact on the patient may provide helpful information for the management of the disorder. Trigger factors, including stress, sleep changes, fasting, caffeine consumption, anxiety, neck pain, and light stimulations, are more reported among people with migraine than other factors and are worth being considered by patients [27,34]. In addition, patients with severe migraine, those with a higher frequency of headaches, or migraine patients who are refractory to medications are highly recommended to evaluate their trigger factors as much as possible [27,35]. On the other hand, the “coping with the triggers” strategy is also suggested because some triggers cannot be avoided and should be approached [36]. In addition, prevention of all exposures can be a source of stress by itself and may restrict daily living. Moreover, sensitivity to some triggers may increase after short exposures and decrease after more exposure. For example, stress and anxiety are highly common, and escaping from them may increase the effect of these triggers because the patient cannot avoid all the sources. In these cases, avoidance may be helpful if possible; otherwise, migraine patients should learn how to deal with these situations [36].

### 3.1 Identifying environmental factors

Different environmental factors are identified and reported to be associated with the initiation of a migraine attack. Table 2 presents some major environmental triggers. Weather and its changes, including decreased atmospheric pressure, low temperature, and high humidity, are triggers in some individuals with migraine. The pathophysiological mechanisms for the association of these triggers with migraine are not clear. However, the release of neuropeptides such as substance P or CGRP after triggers like low barometric pressure is suggested [37,38,39]. Some evidence has revealed that exposure to low atmospheric pressure which can occur during climbing, ascent to high altitudes, or aeroplane travel, needs to be accompanied by other trigger factors to act as a trigger [40]. Although air travel may cause migraine attacks due to atmospheric pressure changes, there are several other factors for travelling, such as poor air conditioning, uncomfortable seats, diet changes, and sleep disturbances, which can trigger headaches. Similarly, climbing is associated with different triggers, including hypoxia, exposure to cold weather, high physical activity, sleep changes, and food problems [38,40]. Although migraine headache attacks might be triggered by increasing the altitude and decreasing oxygen levels, it is worth remembering that according to ICHD-III criteria, “headache attributed to aeroplane travel” is defined as a different disease and should always be considered [25,41,42].

Several studies have evaluated hypersensitivity to light in migraine patients. Although the pathophysiological basis for this phenomenon is not well-recognised, migraine patients are known to be more sensitive to light during the attacks and between them (in the interictal period). It should be mentioned that although bright and flickering light is reported to be a migraine trigger, its effect is not fully evaluated. Long-term exposure, usually discussed as seasonal variations in exposure, is related to a higher attack frequency in migraine patients. Accordingly, photostimulation is an attack trigger in some migraine patients, but even the patients who report it as a trigger may not develop a headache attack after every exposure, which may be because other triggers should be present simultaneously or a certain level of susceptibility is required [27,38,43].

Sensitivity to odours is common, and reportedly females may be more sensitive than males [44]. Some people with migraine are sensitive to odours during and between attacks, which can trigger their headache attacks. Perfumes, cigarette smoke, and cleaning products are among odours with headache triggering properties [27,38,43]. Both nicotine and its withdrawal can trigger headache attacks; therefore, quitting gradually is recommended for the patients [27]. Some legislation, such as banning indoor smoking and avoiding using perfume in the workplace of sensitive patients with migraine, may be protective and helpful [43].

Noise and auditory discomfort are other migraine triggers. Severe and chronic exposure to some types of noise, including traffic and neighbourhood noise from roads, railways, parking cars, and even staircases and footsteps, are associated with an increased risk of migraine [43]. Different patients have different sensitivity levels to these factors; therefore, it is difficult to document and confirm these relationships. Furthermore, more studies are required to reveal the associations and establish guidelines. At this level, the best recommendation for patients is to rely on their personal experiences (using a diary for identifying important triggers might help), avoid the trigger factors if possible or cope with them [38].

### 3.2 Sleep modification

Sleep disturbances are one of the most prevalent migraine triggers. Sleep problems, including insomnia, changes in total sleep time (too little or prolonged), inappropriate timing, and poor sleep quality, such as sleep disruption, early morning awakening, and falling asleep troubles, are associated with a higher frequency of attacks. In addition, changes in the duration and time of sleep on weekends or vacations are well-known triggers of migraine attacks. On the other hand, sleep is known to be a headache relief factor in some migraine patients. The association between headache and sleep changes is bidirectional, which means sleep problems trigger headaches, and headaches may cause sleep disturbances [45,46,47,48].

According to these associations, education and lifestyle changes such as acceptable sleep hygiene, sleep pattern management (regular sleep pattern), and its quality seem to be highly important in preventing migraine headache attacks [13,26]. Good sleep hygiene practices are associated with a lower frequency and duration of headache attacks in migraine patients [49]. Convenient bedrooms with fewer stimulations such as television, cell phone, light, and noise and waking at a specific time range in the mornings, even on weekends, are recommended [26,48]. Filling a sleep diary in parallel with a headache diary will benefit the patients. They may obtain more information about the relationship between headache episodes and their sleep pattern and disturbances. In addition, sleep quality can be improved by reducing stress and management of depression, which are also two modifiable risk factors for chronic pain. Furthermore, the management and treatment of some sleep disorders, such as insomnia and obstructive sleep apnoea, have a positive effect on the prevention of migraine attacks [45,48]. For example, cognitive-behavioural therapy for insomnia is associated with reversing chronic migraine (headache attacks ≥15 days per month) to episodic migraine. Likewise, sleep disturbances and snoring, independently associated with the progression of episodic migraine to chronic migraine, should be treated for better management of migraine. There are some non-modifiable risk factors for the progression of episodic migraine to chronic migraine, such as old age, female gender, low income, and genetics. These underlie the importance of managing modifiable factors, including sleep problems [35,45,46,48,50,51].

Calhoun and Ford recommend a five-component intervention to migraine patients [50], including (a) scheduling consistent bedtime that allows 8 h in bed, (b) eliminating watching television, reading, and listening to music in the bed, (c) using visualisation techniques to shorten time to sleep onset, (d) consuming supper ≥4 h before bedtime and limiting fluids within 2 h of bedtime, and (e) discontinuing naps during the days. This intervention is proven to be quite effective in establishing proper sleep hygiene.

### 3.3 Dietary modification

As mentioned earlier, diet is an important factor in managing migraine. Accordingly, diet components and their triggering effect on migraine attacks should be considered. Several points can contribute to dietary lifestyle modification, including preventing hunger and fasting, having regular meals, sticking to frequent meals (e.g. five or six small meals per day), avoiding some specific food items (mentioned in the following), consuming foods that provide a stable level of blood glucose (slow-digesting foods), cooking food rather than eating processed or fast foods, remembering proper fluid intake and hydration, and having a low-fat diet [52,53,54,55]. The mechanism of associations between the brain and gut (gut–brain axis) is unclear. However, inflammatory factors, the microbial profile of the gastrointestinal system, neuropeptides, serotonin pathway, or stress hormones can impact the association of the brain and gut [56].

Some food items that commonly work as migraine triggers include red wine and alcohol, chocolate, caffeine in products such as coffee, tea, cola, chocolate, and some drugs, processed food, some seafood and fish, ice cream, foods containing nitrates such as bacon, hot dog, ham, and salami, foods containing tyramine such as aged cheese, cheddar cheese, beans, smoked fish, non-fresh meat, citrus fruit, avocados, bananas, and onions, and foods containing monosodium glutamate, which is used as a flavour enhancer in many different kinds of foods such as dried soups, salad dressing, processed foods, some sauces, snack foods, and canned or frozen food items. However, each item might or might not trigger one person, and recording the possible stimulating factors helps the patient with migraine find the offenders. For caffeine consumption, attacks occur, especially due to its withdrawal in chronic consumption or its rebound effect in over usage [27,38,52,54,57,58,59]. The daily consumption of caffeine is recommended to be lower than 200 mg [60]. Although the existing evidence supports the effect of different diets on migraine characteristics, there is no specific diet recommendation for migraine patients [27,52].

A food diary will help the patients find the most important food triggers. Patients may avoid the important food triggers according to their self-experience. In case of no improvement after avoidance or for certainty, patients can start eating the items one by one and check the consequences. The triggering effect is different from one individual to another, and there is no one-size-fits-all diet for all migraine patients. Finally, these triggering effects are associated with dosage, the timing of exposure, and individual and genetic differences [27,38,57,61].

### 3.4 Doing regular physical activity

Physical activity, especially prolonged exertion and fatigue are potential triggers of migraine attacks. Migraine exacerbation by physical activity is part of migraine definition by the International Headache Society. The effect of physical activity on migraine pain is not clear and needs more evaluation. However, individual differences are highly important factors in this association. It is clear that a combination of this trigger with other triggers, such as fasting and dehydration, may increase the chance of headache attacks [62,63]. On the other hand, regular exercise is reported to have protective effects on migraine and some comorbid diseases such as depression, anxiety, obesity, sleep problems, and some other pain disorders such as fibromyalgia [62,64,65].

Although individuals with migraine are advised to avoid exercise during their headache attacks, it is recommended that migraine patients do regular and moderate exercise, especially aerobic exercises such as cycling and walking, after careful warm-up [20,62,66]. The protective effect of regular exercise might be because of its impact on changing the threshold of migraine attacks [64]. Furthermore, migraine patients should reduce sedentary activities such as watching television and increase some activities such as walking or cycling to work, taking stairs and taking a break from sitting for a long time. Additionally, as aerobic exercise is shown to be effective in several disorders, particularly cardiovascular diseases, it is necessary to advise this sort of exercise to migraine patients. Moreover, patients should remember that they may experience no improvement at the beginning, but it is important that they continue to exercise because tolerance to exercise-induced pain occurs gradually [20,62,64,66,67,68]. Furthermore, warming up before exercise, proper nutrition and hydration, adjusting the duration, intensity, and frequency of exercise based on the headache properties and selecting the best type of exercise according to the patient are also recommended [66,69,70].

### 3.5 Management of stress/anxiety

Stress and anxiety are associated with migraine and are common comorbidities [71]. It is a bidirectional relationship, so stress and anxiety can result from frequent and severe headaches, or they may be a trigger factor for provoking headache attacks in migraine patients. The frequency and severity of stressful events and the patients’ judgment about them are important contributing factors in this relationship. Furthermore, stress is an important risk factor for the progression of episodic migraine to the chronic type [6,29,72]. Stress is any stimulus (physical or psychological – internal or external) that starts a biological reaction and results in changing body balance (homeostasis) to adapt [73].

Stress occurs in situations requiring considerable adaptation. There are different types of stressors: death, divorce, loss of beloved ones, losing a job, illness, fatigue, confronting demanding duties, and even minor daily hassles and stressful life events. In addition, fear of probable migraine attacks is another source of stress and anxiety in migraine patients. These events require adaptation and will impact body homeostasis. Some evidence has shown that migraine attacks may occur during the relaxation period after a stressful event. For example, stress in the afternoon may lead to a headache attack in the evening or during the night, and stress in the evening may cause an attack in the morning [30,31,72,74].

It is impossible to avoid all stress sources. Furthermore, avoiding all stressors will be a source of stress, as some will be out of control or unavoidable. According to evidence, the following methods and skills are recommended: stress management techniques, learning to cope with stressors, problem-solving, social support, biofeedback, relaxation training (such as muscle relaxation), changes in living situations and lifestyle, exercise, and avoiding certain situations that cause severe stress and anxiety [27,36,72].

### 3.6 Identifying medications associated with migraine attack

The following medications are associated with triggering migraine attacks: nitrates, theophylline, reserpine, nifedipine, indomethacin, and cimetidine [26,75]. Hormonal contraceptives can trigger migraine attacks; however, their appropriate use can be helpful in the management of some types of migraine, such as oestrogen withdrawal headache, by maintaining a steady level of oestrogen in the serum [26,76].

Excessive intake of acute medications is a risk factor for the development of MOH, which occurs following the consumption of simple analgesics or non-steroidal anti-inflammatory drugs for 15 or more days per month or consumption of opioids, mixed analgesics, triptans, or ergot alkaloids for 10 or more days per month or for more than 3 months [13,77]. MOH is associated with chronic migraine and should be prevented. The following reasons are related to overuse of drugs: being obsessive about taking drugs, fear of pain, anticipatory anxiety, psychological drug dependence, and presence of psychiatric comorbidities, including anxiety [78,79]. Patient education, treatment of frequent or severe prolonged migraine attacks by prophylactic drugs, management of comorbidities, and relaxation treatment can be useful for MOH prevention and treatment [79,80]. Psychological interventions and using behavioural techniques to overcome anticipatory anxiety will help during the treatment period of MOH [81]. Furthermore, MOH should be managed by discontinuing overused drugs (inpatient or outpatient depending on the patient’s condition, type of overused medications, comorbidities, and adequate social support), initiating preventive medications, using a supportive symptomatic treatment and bridging therapy if necessary, and employing psychological support [13,79].

### 3.7 Hormonal factors

Migraine is nearly three times as prevalent in females as it is in males [82]. About 35–50% of females with migraine are likely to experience headache during the premenstrual period, which is associated with decreased plasma levels of oestrogen (after a period of high serum levels) [83,84]. Accordingly, in some patients, migraine attacks may reduce during pregnancy (not in all patients), especially during second and third trimesters and escalate again after delivery (lactation has a protective effect and may reduce migraine attacks) [83,84,85]. During menopause, migraine headaches may increase in some patients due to fluctuations, and reduced oestrogen levels. However, migraine headache improves in many people with menopause because of consistent low levels of oestrogen and ending oestrogen cycles [83,86]. Menstruation-related migraine is usually more severe with longer attack durations and more disabling than non-menstrual types and may show a slight response to analgesics [7,31,83,84]. Oestrogen may play a role in the pathophysiology of migraine through different mechanisms such as increasing nitric oxide release and regulating gene expression and inflammation [87].

Patient education is an important part of the management of hormonal migraine attacks. By informing the patients, they will predict the symptoms, prepare themselves for attacks, and help the physicians control the disease. Patients should be informed about other triggers and avoid as many triggers as possible, especially during the menstruation period. Furthermore, lifestyle modification, such as a good sleep pattern, a healthy dietary pattern and its content, and regular exercise, is highly recommended [83,84,88].

## 4 Weight reduction

Obesity may be associated with a higher frequency of headaches in migraine patients [89,90,91]. Obesity might also be associated with higher severity, and disability of headache attacks in people with migraine, particularly in females in reproductive age [55,90,91,92,93]. The association between migraine and obesity can be modulated via sympathetic dysregulation, adipose tissue function, inflammatory mediators such as CGRP and C-reactive protein, and different neurotransmitters and neuropeptides related to hypothalamic function [55]. Weight loss may reduce the severity and duration of migraine attacks and increase migraine-free days in obese female patients [20,55,94]. Although some evidence supports migraine exacerbation by obesity, the association between obesity and increased migraine risk is still unclear [55]. In addition, obesity and being overweight increase the risk of the progression of episodic migraine to chronic migraine [95,96]. This relationship can be bidirectional, and some patients with chronic migraine may be at a higher risk of gaining weight due to the side effects of some preventive medications and the sedentary lifestyle caused by their pain [91]. On the other hand, no association was reported between the prevalence of migraine and obesity in older patients [97]. It should be mentioned that some evidence shows that being underweight has the same effect like obesity and is associated with a higher risk of migraine compared to normal-weight patients [96,98].

Weight reduction to restore an ideal body weight might be a useful intervention, to control migraine attacks, especially in obese patients [20,55,94]. Behavioural weight reduction is the first line of obesity treatment. Lifestyle modification, including healthy eating habits and physical activity, are highly recommended to achieve this goal. Counselling with a nutritionist to receive an individualised diet, aerobic exercise, education, learning the necessary skills, goal setting, and self-monitoring are important in this regard. Different skills, including healthy cooking methods and the ability to refuse food offered by others, are worth learning.

Additionally, weight should be carefully monitored, especially during migraine prophylaxis, because weight may change as a side effect of some drugs [13,20,52,55,68,99]. Furthermore, attention should be paid to some sleep disorders, such as obstructive sleep apnoea, which is a common consequence of obesity and is associated with the progression of episodic migraine to chronic migraine (polysomnography can help with the diagnosis) [55,100,101,102], and short sleep duration, which is strongly associated with obesity and is related to a higher frequency and severity of migraine attacks [55,103]. In these cases, treatment of both obesity and the associated disorders will result in better management of migraine [55,68].

## 5 Management of comorbidities

Migraine is associated with several different diseases, including psychiatric (e.g. depression, anxiety disorder, bipolar disorder, and panic), cardiovascular (e.g. coronary artery disease, hypertension [controversial] and patent foramen ovale [not enough data to support the improvement of migraine after closure of the patent foramen ovale]), neurological (e.g. epilepsy [controversial]), and some other disorders such as sleep disorder, restless leg syndrome, chronic pains, and asthma. In addition, the association between migraine and hypothyroidism and some gastrointestinal disorders, including irritable bowel syndrome and celiac disease, is reported in the literature. The exact reason for the mentioned comorbidities is unclear. However, it is mentioned that the combination of different disorders may lead to the reduction in activation threshold of different brain parts, change in thalamocortical network excitability, and alteration of organs’ inflammatory state and energy requirement. These changes might lead to activation of the trigeminovascular system. To achieve the best treatment for migraine, comorbid disorders should be considered. In addition, remembering this will help check any potential drug interactions, understand complex clinical pictures, and use single drugs to treat migraine and associated disorders [13,24,30,56,67,104,105,106,107,108,109].

## 6 Behavioural treatment, lifestyle modification, and biofeedback

Behavioural treatment, including relaxation techniques, stress management, cognitive therapy, and biofeedback, is effective in migraine prevention and control and has a strong level of recommendation. Several methods, such as biofeedback-assisted relaxation, mindfulness-based stress reduction and cognitive therapy, muscle relaxation (it is impossible to experience tension and relaxation together), and desensitisation by imagining anxiety stimulants, can be utilised. In addition to this, learning the required skills to reorganise maladaptive thinking patterns, inducing relaxation by diaphragmatic breathing, improving patients’ belief in their abilities, learning problem-solving skills, and some other methods such as yoga, prayer, music therapy, and meditation can be used as well [13,15,30,110,111,112,113].

Behavioural interventions can be offered in private or group sessions. The required techniques may be taught by treatment teams, including different specialists, or by telephone. Public health interventions in the school or workplace and via the Internet and mass media may be helpful. It is important to mention that learning the methods is not enough and practising them is essential for acquiring an effective automatic reaction. To improve the lifestyle and achieve proper sleep hygiene, weight control, suitable dietary habits, and adequate physical activity, behavioural interventions, and education are highly important and should be advised by the physicians. Improving self-awareness and keeping a stable and healthy lifestyle is one of the main aims of behavioural migraine management [15,24,26,55,68,111,114,115]. Biofeedback is a behavioural approach to pain management. According to the previous studies, this method is effective in migraine treatment because it improves control over reactions and enhances one’s awareness of involuntary body functions. The method’s effectiveness is evident in learning to control involuntary body functions by changing circulation (e.g. rising finger temperature, rising forehead temperature, and superficial temporal artery pulsation), decreasing muscle tension, and neurofeedback [15,24,116,117,118]. Thermal biofeedback with relaxation training and also electromyographic biofeedback are reported to have grade A evidence for migraine prevention [15,119]. It reduces the main symptoms of migraine, especially the duration and frequency of headache attacks. The problem with this therapeutic method is the limited number of available specialised centres and certified members and physicians for performing biofeedback. One reason for this problem might be insufficient knowledge of primary care physicians [15,24,117,120].

## 7 Improving patients’ knowledge

The first step for managing a newly diagnosed migraine patient is education. The patients’ knowledge of the disease and its characteristics should be improved, and they should learn that migraine is a chronic disorder that cannot be cured, but it is controllable, and its attacks can be prevented. Furthermore, trigger factors and comorbidities should be discussed with patients while reassuring them of their brain structure and lack of a brain tumour, infection, or other serious illnesses. Describing pharmacological and non-pharmacological strategies for preventing and treating attacks, underlining the difference between abortive and prophylactic drugs, and explaining the prophylactic treatment criteria are essential parts of migraine management. In addition, MOH, potential adverse effects of drugs, and drug interactions should be discussed and considered in migraine management. The patients should be involved in their treatment via education, which may improve migraine management [15,24,83,121].

According to a systematic review study by Kindelan-Calvo et al., therapeutic patient education (mainly focusing on coping strategies to decrease stress and increase relaxation) improves the associated disability, quality of life, and frequency of migraine in migraine patients [122]. However, patients’ education is not enough, and the patients should be willing to change their lifestyles. They should be aware that they can make the required changes and that the changes are extremely helpful [67,121]. The methods and information presented in this article will be effective to be discussed with people with migraine. Finally, to increase the effectiveness of education, the following points were recommended by Rains et al. [123]:

- Limiting educational subjects to up to four topics in each session
- Using every day and straightforward language for education
- Using written supplements to improve the efficacy
- Involving family members in patient education
- Asking for feedback from participants and repeating major points

## 8 Keeping a headache diary/calendar

A headache diary may be helpful for recording attack characteristics, and diagnosis of migraine and its re-evaluation in future visits. And headache calendar will help with follow-ups, treatment evaluation and checking associated factors [124,125]. Recording the frequency, duration and severity of attacks, date of onset and exacerbation, associated symptoms, sleep pattern and its association with the disease, drugs used by the patient and their effects, different triggers (e.g. diet, exercise, stress, and weather), and the effect of menstruation are undoubtedly effective in making an accurate diagnosis, planning the best management strategy, predicting future attacks, improving patient–physician communication, and assessing treatment outcome [13,28,35,57,67]. Recording headache characteristics and associated symptoms and triggers improves the patients’ knowledge of different trigger factors, makes them more familiar with their disease and is useful for disease control and prevention [69,126]. Various paper-based and electronic diary forms have been developed to help patients. Additionally, these tools have limitations like recall bias and difficulties with keeping a daily diary [28,29].

## 9 Using neuromodulation techniques

Based on the available evidence, stimulating the peripheral or central nervous systems can be used to manage migraine. The stimulation can be delivered by electric currents or via changes in a magnetic field around the nervous system. This therapeutic effect may be due to their final impact on cortical or trigeminovascular system activity [23,127,128].

Several different non-invasive and invasive interventions are mentioned in the literature. Non-invasive neuromodulation techniques include transcranial direct current stimulation (tDCS), single-pulse transcranial magnetic stimulation (sTMS), transcutaneous cranial nerve stimulation such as Cefaly^®^ (supraorbital nerve stimulation), non-invasive vagus nerve stimulation (nVNS) such as gammaCore^®^, percutaneous mastoid stimulation, and non-painful brachial electric stimulation. Invasive neuromodulation techniques like occipital nerve stimulation, sphenopalatine ganglion stimulation, and high cervical spinal cord stimulation are among the modalities suggested for treating migraine. Invasive neuromodulations should be considered after all other approaches are tried, and no desired result is achieved [23,127,129].

In general, neurostimulation techniques are reported to be safe. Cefaly^®^ and sTMS have level A evidence, tDCS level B, and nVNS level C [130]. We recommend people with migraine discuss it with their doctor to see if they benefit from these interventions. Neurostimulation techniques can be used as an alternative or in addition to medications, especially when there are contraindications or poor responses to other treatments. This technology might be a safe approach during pregnancy and lactation. However, more studies are needed [16,129].

## 10 Considering dietary supplements

By definition, dietary supplements (nutraceuticals) are food parts that can improve health. Riboflavin (vitamin B2), coenzyme Q10 (CoQ10), magnesium, butterbur, feverfew, and omega-3 polyunsaturated fatty acids are among the dietary supplements suggested for the management of migraine. The effect of these supplements on migraine might be due to their impact on improving brain metabolism, as unstable brain metabolism is suggested in migraine [128,131,132].

Based on some guidelines, 400 mg daily riboflavin, 300 mg daily CoQ10, or 400–600 mg daily magnesium can be used to manage migraine, especially for people with chronic migraine. These supplements are safe and associated with a low rate of adverse events. However, more research is needed on the efficacy and safety of these supplements and on their interactions with medications [23,131,133]. Magnesium and riboflavin received level B (probably effective) and CoQ10 received level C (possibly effective) of evidence from the American academy of neurology and the American headache society [134]. These supplements received a level C recommendation from the European federation of neurological societies [135].

## 11 Acupuncture

Acupuncture is a part of Chinese traditional medicine. Several pieces of evidence support the benefit of acupuncture in managing chronic pain and migraine. Although acupuncture is reported to be effective on both episodic and chronic migraine, the prevention of episodic migraine using acupuncture is supported by more studies and has a strong level of evidence. In addition, the management of acute migraine attacks using acupuncture needs more evaluation. Acupuncture is a safe method and has a low rate of side effects. However, pregnant migraine patients and people with bleeding problems should be careful about using acupuncture and should discuss it with their doctor. The mechanism behind acupuncture’s effect on migraine is unclear, and it needs several treatment sessions to show efficacy [113,136,137].

## 12 Miscellaneous

To achieve better migraine control and prevention, there are some more non-pharmacological recommendations, including:

- Establishing effective communication between patients and health care providers to improve patient satisfaction, adherence to treatment, and treatment outcomes and to reduce the risk of malpractice [15,24].
- Seeking social support, including receiving care and attention from family and spouse and improving family communications [15,138].
- Evaluating the patient’s workplace and occupation; for example, the prevalence of headache is reported to be higher in computer users than controls [43].
- Evaluating the patient’s posture; for example, prolonged cervical flection or hyperextension with frequent head rotation in some patients, such as computer operators, may result in head and neck pain [43].
- Using equipment such as sunglasses, especially when exposed to direct sunlight (overuse of sun-glasses may result in more sensitivity and photophobia), head coverage to avoid exposure to direct sunlight, especially in the summer, cold weather, and wind, fluorescent light filters to have natural lighting, anti-glare filters when using a computer, sound absorption panels, ergonomic chairs at work and when using computers, etc. [27,38,43,139].
- Avoiding some athletic equipment, such as poorly fitting mouth guards and tight helmets [63].

## 13 Conclusion

Although acute and prophylactic pharmacological treatments are used to manage migraine, non-pharmacological approaches are gaining popularity among physicians and patients. A summary of the non-pharmacological points is reported in Table 3. It is essential to identify, avoid, or learn to cope with trigger factors of migraine attacks, including environmental factors (e.g. high altitude, heat, light, sound, odour, and weather and pressure changes), sleep pattern changes (i.e. irregular, too much, too little, and lack of sleeping), diet (e.g. fasting and hunger, irregular meals, dehydration and some food items), physical activity (i.e. fatigue and prolonged exercise), stress and anxiety, some medications, and hormonal changes. Weight control, lifestyle modification, behavioural treatment biofeedback, patient education, improving the patients’ knowledge of the disease, and using headache diaries are other practical non-pharmacological approaches to migraine management are highly recommended. Furthermore, employing neuromodulation techniques, dietary supplements such as riboflavin, coenzyme Q10, and magnesium, and acupuncture can be helpful. Some benefits of non-pharmacological management of migraine are as follow: preventing or reducing the chance of MOH and adverse event of medications, reducing the cost of medication use, helping people with migraine who do not benefit sufficiently from medications or do not want to take them, managing migraine during pregnancy and lactation or when there are contraindications for medication use and improving the efficacy of abortive and preventive treatments (Table 1). Finally, pharmacological and non-pharmacological approaches should not exclude each other, instead, both should be considered.

**Table 3:** Suggested non-pharmacological points for migraine management

Management of/coping with migraine trigger factors
Weight reduction
Management of comorbidities
Behavioural treatment, lifestyle modification, and biofeedback
Improving patients’ knowledge
Keeping a headache diary
Using neuromodulation techniques
Considering dietary supplements
Acupuncture
